# Carbazole Derivatives Binding to Bcl-2 Promoter Sequence G-quadruplex

**DOI:** 10.3390/ph17070912

**Published:** 2024-07-09

**Authors:** Agata Głuszyńska, Joanna Kosman, Shang Shiuan Chuah, Marcin Hoffmann, Shozeb Haider

**Affiliations:** 1 Department of Bioanalytical Chemistry, Faculty of Chemistry, Adam Mickiewicz University, Uniwersytetu Poznańskiego 8, 61-614 Poznań, Poland; joanna.kosman@amu.edu.pl; 2 Laboratory of Molecular Assays and Imaging, Institute of Bioorganic Chemistry, Polish Academy of Sciences, Noskowskiego 12/14, 61-704 Poznań, Poland; 3 School of Pharmacy, University College London, London WC1N 1AX, UKshozeb.haider@ucl.ac.uk (S.H.); 4 Department of Quantum Chemistry, Faculty of Chemistry, Adam Mickiewicz University, Uniwersytetu Poznańskiego 8, 61-614 Poznań, Poland; marcin.hoffmann@amu.edu.pl

**Keywords:** Bcl-2 G-quadruplex, 2F8U, carbazole derivatives, spectroscopy, molecular modeling

## Abstract

In this study, we used ultraviolet-visible (UV-Vis), fluorescence, and circular dichroism (CD) techniques, as well as molecular modeling, to probe the interactions between carbazole derivatives and the G-quadruplex structure formed in the promoter region of gene *Bcl-2*. This gene is a rational target for anticancer therapy due to its high expression in a variety of tumors as well as resistance to chemotherapy-induced apoptosis. We employed a sequence with a specific dual G-to-T mutation that may form a mixed-type hybrid G-quadruplex structure in the Bcl-2 P1 promoter region. The three tested carbazole compounds differing in substitution on the nitrogen atom of carbazole interact with the Bcl-2 G-quadruplex by the same binding mode with the very comparable binding affinities in the order of 10^5^ M^−1^. During absorption and fluorescence measurements, large changes in the ligand spectra were observed at higher G4 concentrations. The spectrophotometric titration results showed a two-step complex formation between the ligands and the G-quadruplex in the form of initial hypochromicity followed by hyperchromicity with a bathochromic shift. The strong fluorescence enhancement of ligands was observed after binding to the DNA. All of the used analytical techniques, as well as molecular modeling, suggested the π–π interaction between carbazole ligands and a guanine tetrad of the Bcl-2 G-quadruplex. Molecular modeling has shown differences in the interaction between each of the ligands and the tested G-quadruplex, which potentially had an impact on the binding strength.

## 1. Introduction

The human *B-cell lymphoma-2* (*Bcl-2*) gene is a GC-rich sequence in a major promoter region P1, one of the two main ones, located 1386–1423 base pairs upstream of the translation start site [1]. The overexpression of proto-oncogene Bcl-2 can lead to the formation of various human cancers [2,3,4,5], which results not only in the inhibition of apoptosis, but also causes the chemotherapy- and radiotherapy-resistance phenomena. This makes Bcl-2 a target for cancer therapy [6,7,8,9,10,11,12,13,14]. Simultaneously, a too low expression of the *Bcl-2* gene may lead to various degenerative diseases associated with the increased apoptotic death of nerve cells [15,16,17,18,19,20,21]. A 39-base-pair DNA sequence of proto-oncogene Bcl-2 has G-rich tracts that can form intramolecular G-quadruplexes (G4 DNAs) [22,23,24,25]. Also in the adjacent region, immediately upstream of the *Bcl-2* gene P1 promoter, a new 28-mer G-quadruplex-forming sequence was found [26]. These four-stranded DNA structures are formed by a planar vertical arrangement of G-quartets, that is four guanines, which are favorably affected by hydrogen bonds of Hoogsteen’s type as well as monovalent cations, and they can be stabilized by small organic ligands [27]. The putative G-quadruplex-forming DNA sequences have been found throughout the human genome [28,29], particularly at telomeres [30] and oncogene promoters [31,32]. Attention was also paid to the special role of G4 DNA in cellular processes [33,34]. The existence of G4 DNA and RNA in human cells has been confirmed [35,36,37], and at the same time, it has been shown that G4 is much more widespread in primary tumors compared to normal tissues [38]. Thus, the presence of G4 in the upstream regions of oncogene promoters may affect the regulation of gene expression at transcriptional or translational levels, and G4 can be a potent therapeutic target, especially for cancer. Because the formation and stabilization or destabilization of G-quadruplexes can be induced by small molecules, G4 DNA can be the target of small ligands. Over the past few decades, many effective G-quadruplex-binding ligands have been identified; however, the development of functional molecules continues and remains challenging [39,40,41,42,43,44]. Among them were ligands whose molecular targets were G-quadruplexes DNA formed on oligonucleotides with the sequences of human proto-oncogene Bcl-2 [41,42,43,45,46]. 

In the previous studies, we detailed the synthesis and examined the interaction of three carbazole ligands with G-quadruplexes formed by sequences corresponding to the regions of c-MYC NHE III1 and c-KIT1, as well as by a telomeric sequence in the presence of sodium and potassium ions [47,48,49,50,51]. It was found that the introduction of different substituents on the carbazole nitrogen atom had little effect on the binding capacity to the G-quadruplex, because the binding constant calculated from UV-Vis and fluorescence titration experiments showed a similar G4 DNA stabilization and comparable binding affinity of 10^5^ M^−1^ [48,49,50]. Here, we describe the DNA-binding properties of these three carbazole ligands to the Bcl-2 G-quadruplex as a molecular target, since it is one of the most frequently overexpressed proto-oncogenes in human tumors. For this purpose, we used various spectroscopic techniques such as UV-Vis and fluorescence, and we employed circular dichroism and augmented experimental measurements with molecular modeling. For our study, we selected a thoroughly characterized 23-mer G-rich sequence known to form a hybrid intramolecular G-quadruplex (structure 2F8U in PDB) [22,23].

## 2. Results

### 2.1. Ligands and Oligonucleotide

**Figure 1:**
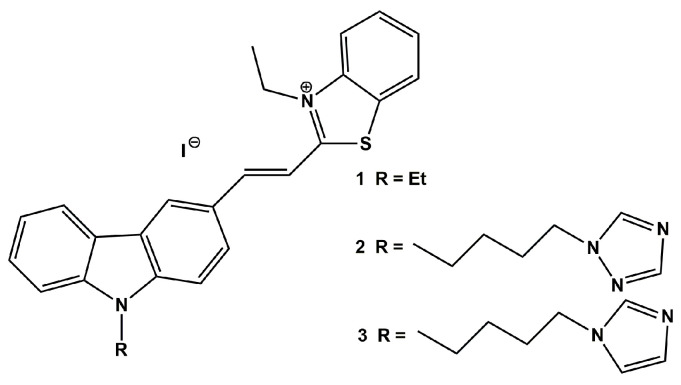
Chemical structures of carbazole ligands **1**–**3**.

The planar carbazole ring of each ligand is linked at the 3-position by a C=C double bond with a cationic benzothiazolium moiety. The ligands at position 9 on the nitrogen atom of carbazole have different substituents: ethyl substituent (ligand **1**) or a longer butyl substituent ended with a triazole ring (ligand **2**) and an imidazole ring (ligand **3**) (Figure 1). Although the chemical properties of both azole substituents do not differ significantly, these substituents may affect the biological activity of the tested compounds [52,53,54,55,56]. Stock solutions of all ligand samples were prepared in DMSO at a concentration of 1.5 mM, preserved at 4 °C, and shielded from light prior to use. The stability of these compounds, monitored over several months, was verified through UV-Vis and fluorescence methods.

**Figure 2:**
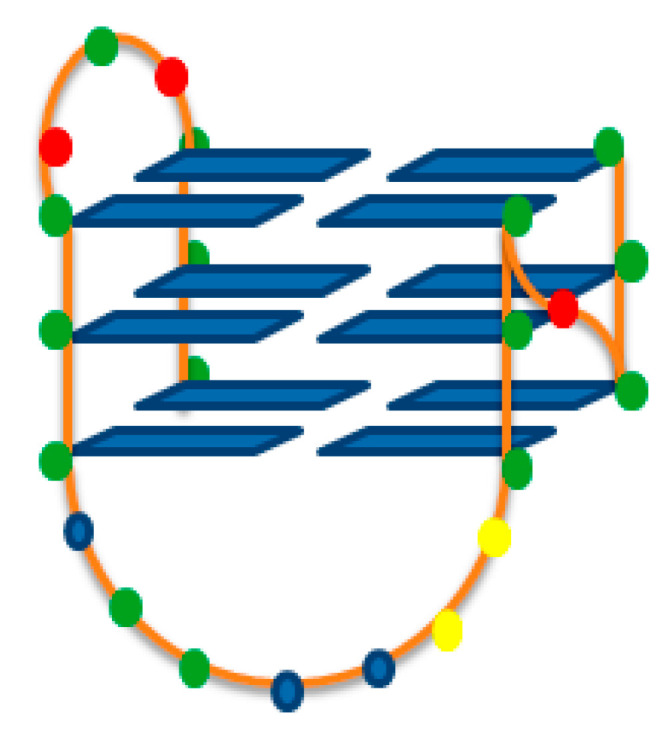
Structure of G-quadruplex formed by Bcl-2 (5′-GGGCGCGGGAGGAATTGGGCGGG-3′) [PDB id: 2F8U]. Guanine, adenine, cytosine, and thymine are represented as green, blue, red or yellow circles.

In our previous reports, we found that the carbazole skeleton is an attractive scaffold of G-quadruplex ligands, but despite the introduction of various substituents on the nitrogen atom, a moderate effect was observed on the G-quadruplex binding capacity and very comparable binding affinity of 10^5^ M^−1^. Although molecular modeling calculations revealed that in the case of ligands **2** and **3**, in addition to end-stacking interactions, enhanced hydrogen bonds between the bound azoles and the bottom of the G-quadruplex groove were also possible, this was not reflected in our experimental results. On the one hand, it could be caused by a too weak participation of hydrogen bonds in the DNA–ligand–G4 interaction, and on the other hand, differences in the PDB id: 2L7V and PDB id: 2O3M structures used for molecular calculations and real structures of G-quadruplexes created during the experiments. Therefore, we decided to check the correlation between molecular calculations and the actual structure of the G-quadruplex during experiments in the case of the biologically important G-quadruplex Bcl-2. This NMR-based structure is formed from the mutant 23-mer with 15,16-G-to-T mutations that in the presence of 100 mM K^+^ forms the hybrid-type G-quadruplex. It contains a core of three G-quartets connected with two lateral loops, containing three and seven nucleotides, and a single-nucleotide double-chain-reversal side loop. The first, third and fourth G-strands are parallel with each other, and the second G-strand is antiparallel with the rest of the strands (Figure 2) [22,23].

### 2.2. UV-Vis Absorption Spectroscopy

To gain knowledge about the interaction between the small carbazole ligands with the G-quadruplex structure 2F8U, visible absorption spectral titrations were performed at first. With the increasing amount of the G-quadruplex, the intensity of the maximum absorption band at about 453 nm decreases, and red shifts of 47, 41 and 42 nm, respectively, associated with 31%, 25% and 27% initial hypochromism, were observed for all three ligands **1**–**3**. With the increasing amounts of the Bcl-2 G-quadruplex, a hyperchromic effect (11–23%) without sharp isosbestic points was observed. The largest spectral changes after the first addition of G4 (~0.1 eq G4) were observed for ligand **1**. After the third addition (~0.3 eq G4), slow hyperchromism was observed for each ligand.

**Figure 3:**
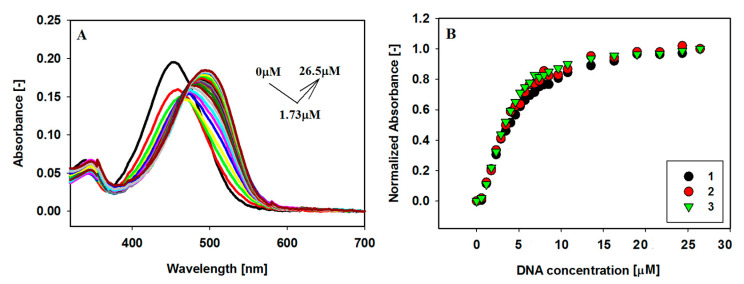
Spectrophotometric titration of ligand **3** (6 µM) with G4 Bcl-2 (0–26.5 µM) in Tris–HCl buffer (10 mM, pH 7.2) containing 100 mM KCl (**A**). Panel (**B**) shows normalized absorbance variations corresponding to the increasing concentrations of Bcl-2 G-quadruplex at 517, 503, 509 nm for ligands **1**, **2** and **3**, respectively.

These significant spectral changes revealed close π–π stacking interactions between the G-quartet and the aromatic carbazole chromophore (Table 1). The absorption spectral changes resulting from the interaction of G-quadruplex Bcl-2 with carbazole ligands **1**–**3** are illustrated in Figure 3 and Figure S1.

### 2.3. Fluorescence Spectroscopy

**Figure 4:**
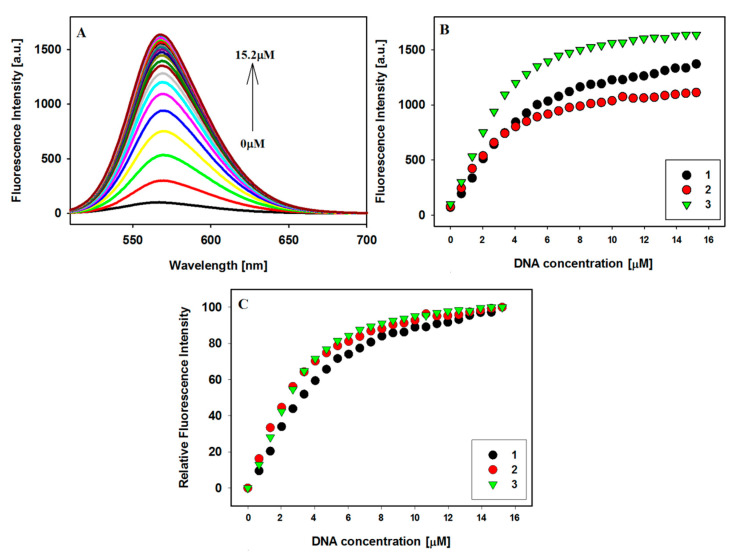
Fluorescence titration spectra of ligand **3** (2 µM) with G4 Bcl-2 (0–15.2 µM) in Tris–HCl buffer (10 mM, pH 7.2) containing 100 mM KCl (**A**); fluorescence binding curves (**B**) and relative enhancement in the fluorescence intensities (**C**) of ligands vs. the increasing concentration of G-quadruplex Bcl-2; λ_ex_: **1**—501 nm, **2** and **3**—494 nm.

The fluorescence technique further supports the external stacking binding mode between three carbazole ligands and the Bcl-2 promoter sequence intramolecular G-quadruplex. The fluorescent properties of carbazole ligands **1**–**3** depend on their chemical molecular structure, but environmental factors also influence the phenomenon of light emission by these compounds. Carbazoles themselves show weak fluorescence in DNA free aqueous solution. The addition of a G4 DNA leads to a significant increase in their fluorescence, and the results of the fluorescence titration experiments are shown in Figure 4 and Figure S2. As we explained earlier, the observed effect may be related to the solvent polarity effect and the subsequent recovery of fluorescence due to the hydrophobic effect of the G-quadruplex [48,49,50].

### 2.4. Binding Stoichiometric Ratios of Ligands to Bcl-2 G-quadruplex

In order to estimate the binding constants of carbazoles **1**–**3** with G-quadruplex, spectrophotometric and fluorescence titration experiments were performed. The spectral data were quantitatively analyzed by constructing Scatchard plots. Although a saturation state was almost reached during the titration experiments (Figure 3B and Figure 4C), due to the complicated interaction of the ligands with G4 DNA (no isosbestic point, initial hypochromicity, and then hyperchromicity and bathochromic shifts), the estimated binding parameters could be subject to a large error. Hence, the spectral data were quantitatively analyzed by creating Benesi–Hildebrand (B-H) plots. For this method, we estimate the value of K_b_ assuming that the stoichiometry of the formed ligand/G4 complex is 1:1. Therefore, the obtained result should be treated as the product of n K_b_ (Table 2) (Figure S3) [57].

### 2.5. CD Spectroscopy

**Figure 5:**
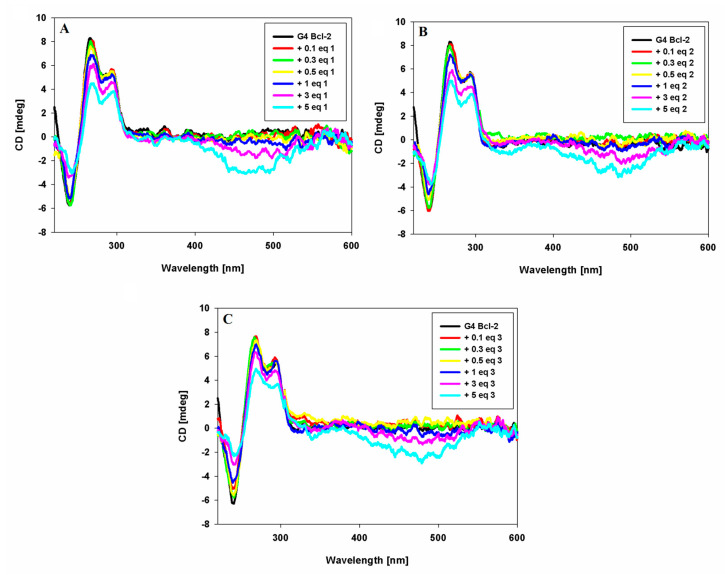
CD spectra of G-quadruplex BCL-2 (5 µM) with increasing amounts of ligands **1** (**A**), **2** (**B**) and **3** (**C**) in Tris–HCl buffer (10 mM, pH 7.2) containing 100 mM KCl.

Circular dichroism spectroscopy (CD) is a useful technique for the characterization of G-quadruplex structures and can be used to effectively distinguish between parallel and antiparallel G4 DNA topologies [58]. CD experiments were carried out to evaluate if the structure of the G-quadruplex is maintained upon carbazole ligands addition. The CD results of the starting DNA structure match with previously published data for the Bcl-2 G-quadruplex promoter sequence [25]. This G4 DNA in the presence of 100 mM K^+^ can form a hybrid-type structure characterized by three intense bands: two positive at 293 nm, 265 nm, and a negative band at 239 nm [27]. Binding by carbazoles does not change the topology of the G-quadruplex, because we still observe bands that are characteristic for this structure. However, we observe a reduction in the intensity of the bands. This decrease in molar ellipticity may be attributed to the interaction between carbazole ligands and quartets of G-quadruplex. This π–π end-stacking interaction is confirmed by the strong negative ICD in the long wavelength absorption region of achiral carbazole ligands (Figure 5).

### 2.6. DNA Melting Studies

**Figure 6:**
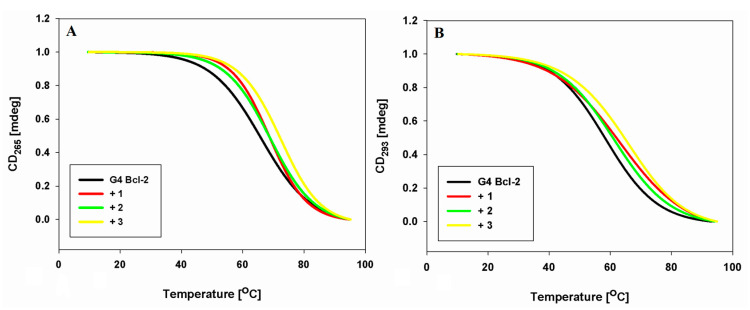
Normalized CD melting profiles of Bcl-2 G-quadruplex at 265 nm (**A**) and at 293 nm (**B**), observed both in the absence and presence of 3 equiv. of ligands **1**–**3** in 10 mM Tris–HCl buffer (pH 7.2) containing 100 mM KCl.

We studied the thermal behavior of the Bcl-2 G-quadruplex in the presence of carbazole derivatives **1**–**3** to observe their conformational changes under the influence of temperature as well as to assess the effect of ligands binding on the stability of the G-quadruplex structure. The CD melting method was used to estimate T_m_ values from the first derivative of the melting curve. Typical melting curves are presented in Figure 6. The experiments were conducted at wavelengths characteristic of the hybrid G4 DNA structure, i.e., at 265 and 293 nm, respectively. In the absence of ligands, the T_m_ value of the G-quadruplex was determined as 65.6 °C and 58.2 °C in solution containing 100 mM KCl at 265 and 293 nm, respectively (Table 3). Ligands **1** and **2** thermally stabilize the G-quadruplex on similar levels (ΔT_m_ = 2.6–3.8 ± 0.1–0.2 °C), while the twice-highest stability of the complex was observed for ligand 3 (ΔT_m_ = 6.3–6.4 ± 0.3–0.4 °C).

### 2.7. Molecular Modeling Studies

**Figure 7:**
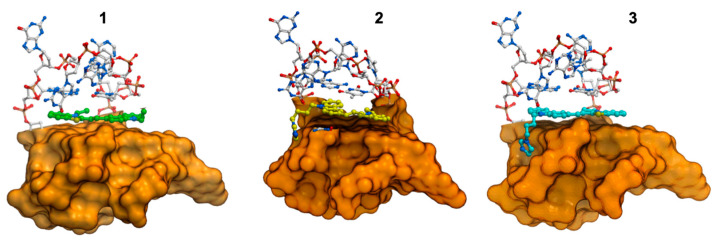
Docked ligands **1** (green), **2** (yellow) and **3** (cyan) on the 5′ external G-tetrad of the hybrid type G-quadruplex. Cartoons represents the side view of the complex. The loop is illustrated as white sticks, and the quartets are drawn in a surface representation.

All ligands **1**–**3** were docked into the grid centered above the 5′ end of G4 (G1–G9–G17–G21). We decided to dock the ligands on this side of the G-quadruplex based on previous research of Haider et al. [59,60]. The most important was the generation of the pseudointercalation binding site between the 5′ terminal tetrad and the loop which allowed docking of the ligands into the chosen space. We did not observe differences between the position of the core (carbazole with benzothiazolium moiety) of our ligands. All of them stack above an external tetrad on the 5′ end of G4 (Figure 7). The ligand stayed in trans-conformation, which allowed good stacking with the G-tetrad. Also, we observed very small differences between the position of the substituent of the triazole and imidazole. Both are directed into the side groove and within hydrogen bonding distance with C4. We selected the most energetically favorable position of the ligands and G-quadruplex as a starting structure for further simulations.

Evaluation of the stability of 2F8U/**1**–**3** complexes was assessed based on the interactions and ligand dynamics. In the most stable conformation of ligand **1**, we observe a small movement of the ligand at the 5′ end where carbazole interacted by end-stacking mostly with three guanines G1, G9 and G21. As a result of this movement, the ethyl substituent on the benzothiazolium ring rotates toward the loop and is sandwiched between the ribose sugar rings of T15 and T16 to avoid any steric clashes. There is considerable movement of ligand **2** during the simulations. The carbazole ring becomes sandwiched between G1 of the terminal tetrad and A13. As the simulation progresses, the ligand exhibits quite good stability at the 5′ end. The ligand interacts by end-stacking mode with the tetrad in particular with three guanines (G1–G17–G21), while the triazole moiety is quite dynamic and interacts with residues in the grooves of the complex. At 1000 ns, we observed that while the carbazole moiety and the benzothiazolium rings are stacked on G1 and G17, respectively, the triazole side chain points outwards into the solvent. In the most stable conformation of ligand **3**, the carbazole ring makes π–π stacking interactions with G1 and G9, and the benzathiazolium ring is stacked on G21. The imidazole substituent makes stable hydrogen bond interactions with the N2 atom of G9. The N4 atom of C4 is also within close proximity of the imidazole ring. These interactions are maintained throughout the course of the simulation and explain the stabilizing effect of ligand **3**, which are in excellent agreement with the experiments. All three ligands stayed in the trans-conformation throughout the simulations.

**Figure 8:**
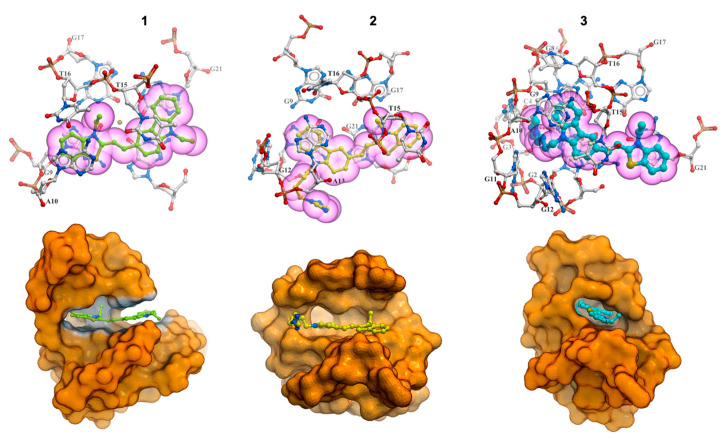
The most stable conformations of ligand **1** (green), **2** (yellow) and **3** (cyan) complexes. The ligands stack on the 5′ terminal tetrad and are sandwiched between T15 from the loop. Additional interactions are observed in the 2F8U/**3** complex. The illustrations in the bottom row highlight the respective binding sites of the ligands (represented as sticks) on the G-quadruplex (surface representation).

The structure of the G-quadruplex in all three complexes was stable over the course of the simulations. Analysis of the interactions between the ligands and the G-quadruplex indicated that all of the ligands can make multiple interactions with the G-quadruplex (Figure 8). Ligand **1** and **3** span and stack over at least three bases on the terminal tetrad, namely G9–G1–G21. Ligand **2** stacks only on G1 and G21. Furthermore, T15 from the loop stacks on top of the ligands. It is interesting to note that while T15 makes π–π stacking interactions with the carbazole moiety in ligand **1** and **3**, it stacks atop the benzathiazolium ring in ligand **2**. Additional interactions between the imidazole and triazole side chains differentiate between ligands **1** and **2**/**3**. Moreover, the A10–T15 base pair is lost in 2F8U/**1**,**2** complexes, while it is retained in the ligand **3** complex. These additional interactions observed in the 2F8U/**3** complex impart further stability to the complex, thereby explaining why ligand **3** possess the best stabilization properties of the three ligands.

**Figure 9:**
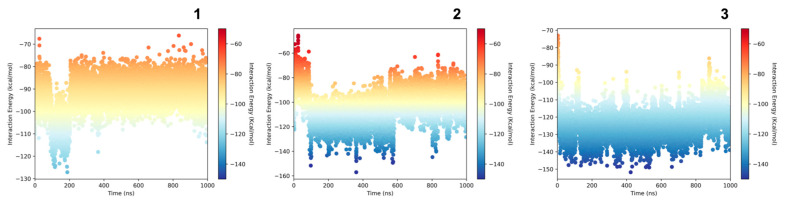
Interaction energy computed from the simulations at every frame. The average interaction energy for 2F8U/**1** complex is −92.95 kcal/mol, for 2F8U/**2** complex is −104.36 kcal/mol and for 2F8U/**3** complex is −124.39 kcal/mol.

The computed value of the interaction energy for every frame in the trajectories is presented in Figure 9. We observed the lowest value for complexes 2F8U/**3** (average value −124.39 [kcal/mol]), for 2F8U/**2** (average value −104.36 [kcal/mol]) and 2F8U/**1** (average value −92.95 [kcal/mol]). The calculated energies indicate that ligand **3** bound much stronger to the G-quadruplex in comparison to ligands **1** and **2**. These observations are in agreement with our experimental results. For ligand **1**, it was expected because the ligand does not possess any additional acceptor/donor atom in comparison to ligands **2** and **3**. However, it is surprising in the case of ligand **3**. The only differences between ligands **2** and **3** is the structure of the triazole connected by the butyl chain to the carbazole in ligand **2** and the imidazole structure instead of the triazole in ligand **3**. This small change results in a completely different behavior to the studied G-quadruplex.

## 3. Discussion

The G-quadruplex binders stabilize G-quadruplex structures, in most cases, via π–π stacking of the planar rigid aromatic structure of a ligand on the external tetrad of guanines. The presence of heteroatoms in the aromatic core and/or the side chains of compounds, often endowed with positive charge, not only increases the ligand’s solubility in an aqueous environment but also enables electrostatic interactions resulting in the binding of ligands. Ligand side chains are important structural elements in optimizing their binding mode to G4 DNA. The tested ligands contain a planar element in their structures that can interact with the external G-tetrads of the G-quadruplex (carbazole ring), a heteroatom with positive charge (heterocyclic benzothiazolium moiety) and azole moieties that can be located in the groove, forming hydrogen bonds that increase the stability of the research complexes.

In order to evaluate binding affinity of carbazole ligands to Bcl-2 G-quadruplex DNA, spectrophotometric and fluorescence titration were performed (Figure 3, Figure 4, Figures S1 and S2). UV-Vis absorption measurements indicated a complicated two-step process of interaction of ligands with G4. The initially observed hypochromism (41–47 nm, ligand **1** shows the largest decrease in absorbance) is probably caused by electrostatic interactions of the phosphate groups of G-quadruplex with the positively charged ligands. Increasing the concentration of ligands changes the binding mode to end-stacking interaction (hyperchromicity 11–23%) (Table 1). The fact that we do not observe a sharp isobestic point is probably due to this course of interaction, where we analyze many conformations of quadruplex and quadruplex–ligand complexes in solution. Fluorescence titration experiments confirmed that the tested ligands are stacked at an external quartet of the Bcl-2 G-quadruplex. This is evidenced by the increase in fluorescence intensity of weakly emissive ligands **1**–**3** in the buffer solution under the influence of Bcl-2 G4, which protects the ligand against quenching by polar water molecules. The end-stacking binding mode with external G-tetrads was also confirmed by circular dichroism spectroscopy. The strong negative ICD in the long wavelength absorption region of achiral carbazole ligands below 500 nm indicates that π–π end-stacking interactions could be the dominant interaction mode between compounds **1**–**3** and G4 Bcl-2 (Figure 4). A decrease in CD signal intensity (the position of signals has not changed) was found upon the addition of up to five equivalents of ligands, suggesting the presence of ligand-dependent perturbations in the studied G4 structure. However, this may be the result of the stacking interaction and not the destabilization of G4 [61].

Molecular modeling studies have shown that all of the ligands in trans-conformation stack above the external tetrad on the 5′ end of G4 with good fit. Research has shown that the long loop above the external G-tetrad at the 5′ end of the G-quadruplex (A10–G11–G12–A13–A14–G15–G16) is the most flexible and movable part of the research structure, and that ligand **3** stabilizes this long loop. Simultaneously, the imidazole moiety of this ligand is located in the groove, making hydrogen bonds that increase the stability of the complex. While the results obtained from energy calculations are not unexpected in the case of ligand **1**, where there is no additional acceptor/donor atom in comparison to ligands **2** and **3**, these are surprising in the case of ligand **3**. The only difference between ligands **2** and **3** is the azole ring at the end of the butyl chain: triazole in ligand **2** and imidazole instead of the triazole in ligand **3**. This difference is clearly reflected in our simulation results. The binding parameters calculated from the absorbance and fluorescence titrations are in good agreement at the level of 10^5^ M^−1^ (Table 2). The binding affinities of carbazole ligands for the tested hybrid Bcl-2 G4 DNA structure were very comparable and were in the ranges of 2.2–3.8 × 10^5^ M^−1^ and 2.5–4.7 × 10^5^ M^−1^, for UV-Vis and fluorescence experiments, respectively (compound **1**: 2.2–2.5 ± 0.2–0.3 × 10^5^ M^−1^, compound **2**: 2.8–3.6 ± 0.2 × 10^5^ M^−1^, compound **3**: 3.8–4.7 ± 0.2 × 10^5^ M^−1^). Also, the CD spectra did not show any difference in the mode of interaction regardless of the functional substituents in particular carbazole ligands. We did not observe positive ICD signals or isoelliptic points that could indicate the groove binding to the G-quadruplex structure. We obtained additional experimental information about the complexes studied from DNA melting studies. The T_m_ values for all the complexes in the presence of 100 mM KCl at 265 nm and 293 nm were very similar (ligand **1** ΔT_m_ = 3.1–3.8 °C, ligand **2** ΔT_m_ = 2.0–3.0 °C, ligand **3** ΔT_m_ = 6.3–6.5 °C). Nevertheless, the obtained values are in excellent agreement with the order of binding constants and confirm that we are dealing with one hybrid type G-quadruplex, known as (3 + 1) G-quadruplex, and not the coexistence of many G-quadruplexes with both parallel and antiparallel conformations.

We conclude that the investigated ligands stabilize the Bcl-2 G-quadruplex structure under biologically relevant conditions. All analytical techniques used indicated the end-stacking binding mode of carbazole ligands on the external G-tetrad of the Bcl-2 quadruplex. In the case of ligand **3**, we observed a slightly stronger binding affinity, which may be related to the formation of hydrogen bonds between the imidazole substituent and the G-quadruplex groove. This observation is in agreement with our experimental findings.

## 4. Materials and Methods

### 4.1. Ligands

Carbazole derivatives were synthesized by the method reported in our previous paper [47,50,51]. The purity of ligands was examined by the HPLC technique. These studies were performed on a Waters HPLC system 1525 (Waters Corporation, Milfort, MA, USA) equipped with a Photodiode Array detector 2998 and a fluorescence detector 2475, a Breeze interface. A Symmetry^®^C18 column (d_p_ = 3.5 μm, 75 × 4.6 mm i.d.) (Waters) was used with isocratic elution (70% MeOH and 30% 10 mM NaCl, flow rate 1 mL min^−1^).

### 4.2. Oligonucleotide

The quadruplex-forming 23-mer deoxyribonucleotide with the Bcl-2 sequence of 5′-GGGCGCGGGAGGAATTGGGCGGG-3′ (2F8U code in the Protein Data Bank) was purchased from Genomed (Warsow, Poland) and was used without further purification. The strand concentration was determined at 260 nm at 85 °C using an extinction coefficient of 254,600 M^−1^ cm^−1^ as calculated from the published values of molar absorptivities of nucleotides [62]. Before using, the oligonucleotide solution was heated at 90 °C for 5 min and subsequently allowed to slow cooling to room temperature, and then it was stored at 4 °C overnight. Tris Base (CAS Number 77-86-1) and Tris HCl (CAS Number 1185-53-1) were obtained from Aldrich Chemical Co. (Poznań, Poland) and used as received. Milli-Q filtered water (Merck Millipore, Burlington, MA, USA) was used throughout.

### 4.3. Absorption Spectroscopy

The absorption spectra were recorded on a Cecil CE-2021 spectrophotometer (Cambridge, UK) in the 200–700 nm range at 25 °C. All of the measurements were carried out using a 10 mm quartz cell. UV-Vis absorption titrations were carried out by the stepwise addition of small aliquots of 754 µM/strand of G4 DNA solution to a cell containing 1000 µL of 6 µM ligand in buffer. After each addition of DNA, we allowed a three-minute equilibration period. All measurements were performed in a 10 mM Tris–HCl buffer (pH 7.2) containing 100 mM KCl.

### 4.4. Fluorescence Spectroscopy

The fluorescence measurements were conducted using a Jasco FP 8200 spectrofluorimeter (Tokyo, Japan) with the cell compartments maintained at 25 °C. All of the measurements were carried out using a 10 mm quartz cell. The fluorescence spectra were collected from 510 to 700 nm with both excitation and emission slits being 5 nm. Fluorescence titrations were carried out by the stepwise addition of small portions of the 663 µM/strand of G4 DNA Bcl-2 solution to a cell containing 1000 µL of 2 µM ligand in buffer. Three minutes was the equilibration time after each DNA addition followed by emission spectrum recording. The excitation wavelength depended on the absorption spectrum of the ligand. All measurements were performed in a 10 mM Tris–HCl buffer (pH 7.2) containing 100 mM KCl.

### 4.5. Circular Dichroism

Circular dichroism (CD) spectra were obtained using a Jasco J-810 spectropolarimeter (Jasco, Tokyo, Japan) in the spectral range from 210 to 600 nm with a 500 nm/min scan speed and bandwidth of 1 nm. Spectra were recorded in quartz cuvettes of 1 cm path length and averaged from 3 scans. Measurements with oligonucleotide were performed at 25 °C in a 10 mM Tris–HCl buffer (pH 7.2) containing 100 mM KCl. The concentration of 23-mer oligonucleotide Bcl-2 was 5 µM/strand. Ligands were added to G4 DNA solution at increasing concentration from 0.1 to 5 molar equivalents.

In the melting studies, the temperature of the samples was maintained by a Jasco Peltier temperature controlled cell holder. The melting profiles of the Bcl-2 G-quadruplex samples were obtained by heating the 2 μM oligonucleotide solution in 10 mM Tris HCl buffer, pH 7.2, and 90 mM LiCl and 10 mM KCl or 100 mM KCl at 90 °C for 5 min followed by slow cooling and storing at 4 °C overnight. The melting profiles were recorded in the absence and presence of 3 eq of ligands **1**–**3** in the 10–95 °C range with a 1 °C/min scan speed. All experiments were carried out using quartz cuvettes with a 10 mm optical path. Data were collected at 265 nm. Typically, three replicate experiments were performed, and average values are reported.

### 4.6. Ligand–Quadruplex Binding Study

Data from the binding studies, which included spectrophotometric and spectrofluorimetric titrations, were processed using the Benesi–Hildebrand transformation [57]. Experiments were carried out in the same manner—after each G4 DNA addition, the titrated solution was incubated for 3 min followed by the UV-Vis or fluorescence spectrum measurement. The titration process was continued until minimal changes were noted in the absorption or fluorescence spectra with further additions of 2F8U. The Benesi–Hildebrand transformation, which calculates the K_b_ value, incorporates Equations (1) and (2) based on a one-site ligand binding model.

Equations (1) and (2) describe the one-site ligand binding model:

(1) $$\frac{cL}{A-Ao}=\frac{cL}{Am-Ao}+\frac{cL}{\left(Am-Ao\right)nKb}\times \frac{1}{cG4\;DNA}$$

(2) $$\frac{1}{F-Fo}=\frac{1}{Fm-Fo}+\frac{1}{\left(Fm-Fo\right)nKb}\times \frac{1}{cG4\;DNA}$$

where cL is the total concentration of ligands, Ao is the absorbance of ligands in the absence of G4 DNA, A is the absorbance recorded in the presence of added G4 DNA at the concentration of cG4 DNA, Am is absorbance in the presence of added [cG4 DNA]max, Fo is the fluorescence of ligands in the absence of G4 DNA, F is the fluorescence recorded in the presence of added G4 DNA, Fm is fluorescence in the presence of added [cG4 DNA]max, *n* is the number of bound ligand molecules per G-quadruplex, and K_b_ is the binding constant.

### 4.7. Molecular Modeling Studies

The design protocol we used was based on the combined use of docking and molecular dynamics simulations, in which the ligand, the target and the solvent were described by an atomistic force field. In particular, we first used the docking calculations in order to establish the favorable position of the ligands. We then performed molecular dynamics simulations to check the stability of the studied complexes. The final quality measure was the average value of the scoring function on a long molecular dynamics simulation. The sequence from the Bcl-2 (PDB ID: 2F8U, 5′-(GGGCGCGGGAGGAATTGGGCGGG)-3′ promoter region was chosen as a model for the G-quadruplex for both molecular docking of the ligands and molecular simulations [63]. We used the DNA modifications to the AMBER force field for system modeling [64,65,66,67,68]. The topologies were generated by the tleap program in the AMBERTOOLS23 software package [69].

### 4.8. Ligand Docking and Modeling

The hybrid form of the G-quadruplex (PDB id: 2F8U) formed in the human Bcl-2 promoter region was used as a receptor in the docking experiments of ligands **1**–**3**. The pseudo-intercalation ligand-binding site was generated above the 5′ terminal tetrad based on a protocol described by Haider and Neidle [60]. The docking grid was centered on the 5′ end of G4, specifically on G1–G9–G17–G21 and the long loop above (A10–G11–G12–A13–A14–G15–G16). The conformation and position of the loop was changed based on the suggestions of Haider et al. before the docking procedure [59,60]. The G-quadruplex and K^+^ ions were kept frozen in the original NMR structure throughout the docking procedures [22,23]. For each ligand, we used the standard procedure of docking in the ICM Molsoft program [70]. The best position of the ligand was chosen based on the lowest energy ranking.

### 4.9. Molecular Dynamics Simulations

The crystal structure of the intramolecular hybrid G-quadruplex formed from the *Bcl-2* gene promoter region 5′-GGGCGCGGGAGGAATTGGGCGGG-3′ (PDB id:2F8U) was used as the starting structure for the simulation. First, 2 K^+^ ions were added in the central channel between the quartets. The structure was explicitly solvated with TIP3PBOX water molecules in a periodic box whose boundaries extended at least 10 Å from any solute atom. Additional K^+^ counterions were added to the system to neutralize the charge on the quadruplex backbone. The counterions were automatically placed, using the Coulombic potential, into the most negative locations by the LEAP program. Periodic boundary conditions were applied to avoid the edge effect. The particle mesh Ewald (PME) method was employed to calculate long-range electrostatic interactions. The AMBER ff14sb force field [66] with parmbsc1 [67] and χ_OL3+OL15_ [68,69] modifications were applied for G-quadruplexes, ions, and water molecules. The ligands were parameterized using the GAFF force field [65]. A 5000 step steepest descent minimization followed by a 5 ns MD equilibration was performed in which the system was gradually heated to 300 K. During the equilibration, the G-quadruplex and the ligand were restrained, while the solvent and ions were allowed to equilibrate. The production run with no restraints on the system was run for 1 μs (1000 ns) simulations using the ACEMD molecular dynamics engine [71]. The trajectories were analyzed using the tools available in the GROMACS suite (www.gromacs.org, accessed on 4 June 2024) [72] and visualized by means of the VMD program [73], ICM-Pro Molsoft molecular modeling package (www.molsoft.com) [70], and pymol (www.pymol.org).

### 4.10. Interactions and Stability Analysis

To assess the stability of the complexes, we calculated the interactions energies between the G-quadruplex and the ligand complexes. The results allow the correlation of the structural conformations adopted by the G-quadruplex under the dynamic influence of the ligands. The NAMD interaction energy calculation tool implemented in the VMD program [73] was used to estimate the interaction energy. As a result, we obtained a graph of energy dependence on time (Figure 8). The final value is the average value calculated throughout the simulations. 

## 5. Conclusions

We have shown that three carbazole ligands, differing in substituents at the nitrogen atom of carbazole, interact and stabilize the G-quadruplex formed by the DNA sequence derived from the promoter region of gene Bcl-2. As a research tool, we used three spectroscopic techniques, such as UV-Vis spectrophotometry, fluorescence, and CD, as well as molecular modeling calculations. The results have shown that all ligands interact with Bcl-2 G4 by the same binding mode with the very comparable binding affinities in the order of 10^5^ M^−1^. Large spectral changes of ligands at higher G4 concentrations were observed during the absorption and fluorescence measurements. In the first case, hypochromic and hyperchromic effects with a simultaneous bathochromic shift without sharp isosbestic points were observed. In the second case, a strong fluorescence enhancement of the ligand was observed, which was a result of the restriction of ligand freedom (rotation) in a rigid G-tetrad binding site and solvent polarity effect. All these suggested the π–π interaction between the carbazole ligands and a guanine tetrad of the Bcl-2 G-quadruplex, which was additionally confirmed by the induced CD band of the ligand. Molecular simulations and energy analysis also confirmed the end-stacking binding mode. Our experimental results have shown that the introduction of different substituents on the nitrogen atom of carbazole had a moderate and comparable impact on the G-quadruplex binding properties. Theoretical research has shown that both azole substituents in ligands **2** and **3** are directed into the side groove of the G4, but only the imidazole substituent interacts by a hydrogen bond, and that ligand **3** possesses the best stabilization properties of the three ligands. Our experimental results suggest rather weak hydrogen bonding in relation to stronger π–π end-stacking interactions. These discrepancies may be due to differences in the structure of 2F8U adjusted for molecular calculations and the actual G-quadruplex structure formed by the oligonucleotide under experimental conditions.

## Figures and Tables

**Table 1:** Spectral effects for the ligands **1**–**3** bound to G4 DNA.

Ligands	Δλ_max_ [nm] ^a^	Hypochromicity [%] ^b^	Hyperchromicity [%] ^b^
**1**	47	31	23
**2**	41	25	11
**3**	42	27	23

^a^ Δλ batochromic shift. ^b^ Hypochromicity and hyperchromicity were measured at λ_max_. The hypo- and hyperchromicity percentages were calculated using following equations: %Hypo = [(A_f_ − A_b,min_)/A_f_] × 100 and %Hyper = [(A_b,max_ − A_b,min_)/A_b,max_] × 100, where A_f_, A_b,min_, and A_b,max_ are the absorbances of the free ligand, bound ligand with absorbance minimum, and bound ligand with absorbance maximum (excess of G4), respectively.

**Table 2:** Parameters for the interaction of ligands **1**, **2** and **3** with Bcl-2 G-quadruplex determined using Benesi–Hildebrand method in absorption and fluorescence titration experiments (K_b_—binding constant, n—number of bound ligand molecules per G-quadruplex; **1**—λ = 517 nm, λ_ex_ = 501 nm, **2**—λ = 503 nm, λ_ex_ = 494 nm, **3**—λ = 509 nm, λ_ex_ = 494 nm).

Ligands	Benesi–Hildebrand Method, nK_b_ (×10^5^ M^−1^)	
	Spectrophotometric Titration	Fluorescence Titration
**1**	2.2 ± 0.3	2.5 ± 0.2
**2**	2.8 ± 0.2	3.6 ± 0.2
**3**	3.8 ± 0.2	4.7 ± 0.2

**Table 3:** Summary of the DNA melting studies.

Derivative	T_m_/ΔT_m_ (°C) ^a^	T_m_/ΔT_m_ (°C) ^b^
No drug	65.5 ± 0.1	58.2 ± 0.1
**1**	68.6 ± 0.1/3.1	62.0 ± 0.2/3.8
**2**	68.5 ± 0.1/3.0	60.8 ± 0.1/2.6
**3**	71.8 ± 0.3/6.3	64.6 ± 0.4/6.4

^a,b^ T_m_ of G4 Bcl-2 in the absence and presence of ligands **1**–**3** in 10 mM Tris-HCl buffer (pH 7.2) in the presence of 100 mM KCl at 265 nm (a) and 293 nm (b). ΔT_m_ was obtained from the differences in the melting temperatures of the 3 eq. ligand bound and uncomplexed G4 DNA.

## Data Availability

The data are contained within the article and Supplementary Materials. The simulation data can be downloaded from https://doi.org/10.5281/zenodo.12532104, accessed on 4 June 2024. The data include the trajectories and their corresponding parameter file.

## Supplementary Materials

The following supporting information can be downloaded at: https://www.mdpi.com/article/10.3390/ph17070912/s1, Figure S1: Spectrophotometric titration of ligands **1** (A) and **2** (B) (6 µM) with G4 Bcl-2 2F8U (0–26.5 µM) in Tris–HCl buffer (10 mM, pH 7.2) containing 100 mM KCl; Figure S2: Fluorescence titration spectra of ligands **1** (A) and **2** (B) (2 µM) with G4 Bcl-2 2F8U (0–30 µM) in Tris–HCl buffer (10 mM, pH 7.2) containing 100 mM KCl; λ_ex_: **1**—501 nm, **2**—494 nm; Figure S3: Benesi–Hildebrand plots of absorbance (A,C,E) and fluorescence (B,D,F) binding data of ligands **1** (A,B), **2** (C,D), and **3** (E,F) with G4 Bcl-2 2F8U.
