# Supplementary material for: Carbazole Derivatives Binding to Bcl-2 Promoter Sequence G-quadruplex
Source: Pharmaceuticals (Basel). 2024 Jul 9;17(7):912. doi: 10.3390/ph17070912 (PMC11279778; doi:10.3390/ph17070912)
Supplement: Supplementary file 1 [file pharmaceuticals-17-00912-s001.zip › pharmaceuticals-2994189-supplementary.pdf]

# Supporting Information

### Carbazole derivatives binding to Bcl-2 promoter sequence G-quadruplex

Agata Głuszyńska [1,*], Joanna Kosman [1,2], Shang Shiuan Chuah [3], Marcin Hoffmann [4] and Shozeb Haider [3]

[1] Department of Bioanalytical Chemistry, Faculty of Chemistry, Adam Mickiewicz University, Uniwersytetu Poznańskiego 8, 61-614 Poznań, Poland; aglusz@amu.edu.pl (A.G.); joanna kosman@amu.edu.pl (J.K.)
[2] Laboratory of Molecular Assays and Imaging, Institute of Bioorganic Chemistry, Polish Academy of Sciences, Noskowskiego 12/14, 61-704 Poznań; joanna kosman@amu.edu.pl (J.K.)
[3] School of Pharmacy, University College London, London WC1N 1AX, UK; shozeb.haider@ucl.ac.uk (S.H.)
[4] Department of Quantum Chemistry, Faculty of Chemistry, Adam Mickiewicz University, Uniwersytetu Poznańskiego 8, 61-614 Poznań, Poland; marcin.hoffmann@amu.edu.pl (M.H.);

### Contents

**Figure S1.** Spectrophotometric titration of ligands **1** (A) and **2** (B) (6 μM) with G4 Bcl-2 2F8U (0–26.5 μM) in Tris–HCl buffer (10 mM, pH 7.2) containing 100 mM KCl.

**Figure S2.** Fluorescence titration spectra of ligands **1** (A) and **2** (B) (2 μM) with G4 Bcl-2 2F8U (0–30 μM) in Tris–HCl buffer (10 mM, pH 7.2) containing 100 mM KCl; $\lambda_{ex}$: **1**—501 nm, **2**—494 nm.

**Figure S3.** Benesi-Hildebrand plots of absorbance (A,C,E) and fluorescence (B,D,F) binding data of ligands **1** (A,B), **2** (C,D), and **3** (E,F) with G4 Bcl-2 2F8U.

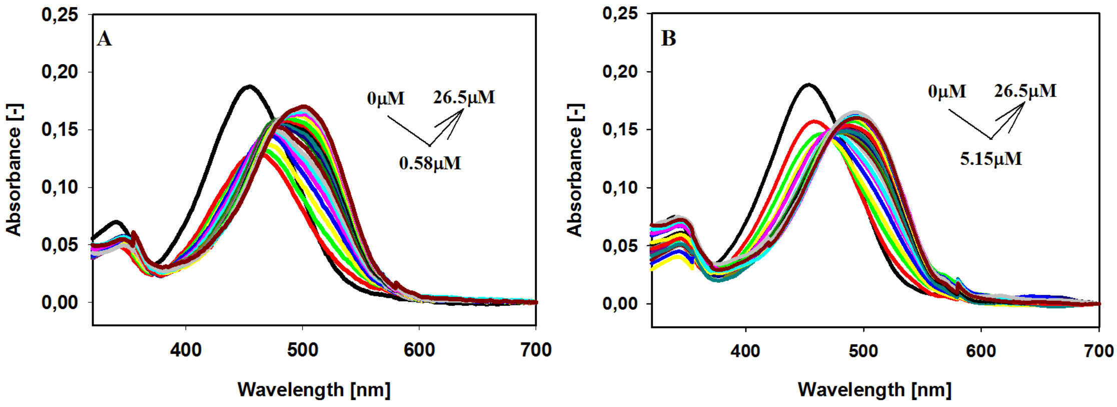


**Figure S1.** Spectrophotometric titration of ligands **1** (A) and **2** (B) (6 µM) with G4 Bcl-2 2F8U (0 - 26.5 µM) in Tris-HCl buffer (10 mM, pH 7.2) containing 100 mM KCl.

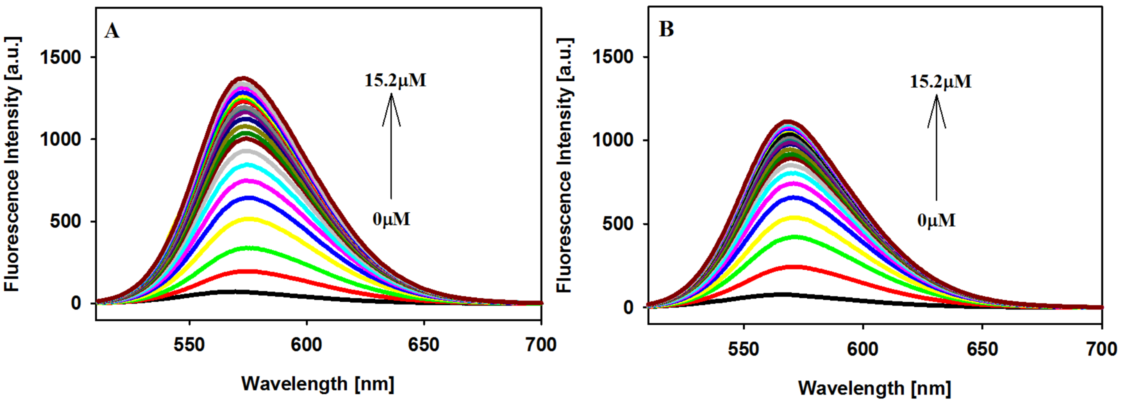


**Figure S2.** Fluorescence titration spectra of ligands **1** (A) and **2** (B) (2 µM) with G4 Bcl-2 2F8U (0–30 µM) in Tris-HCl buffer (10 mM, pH 7.2) containing 100 mM KCl; $\lambda_{ex}$: **1**—501 nm, **2**—494 nm.

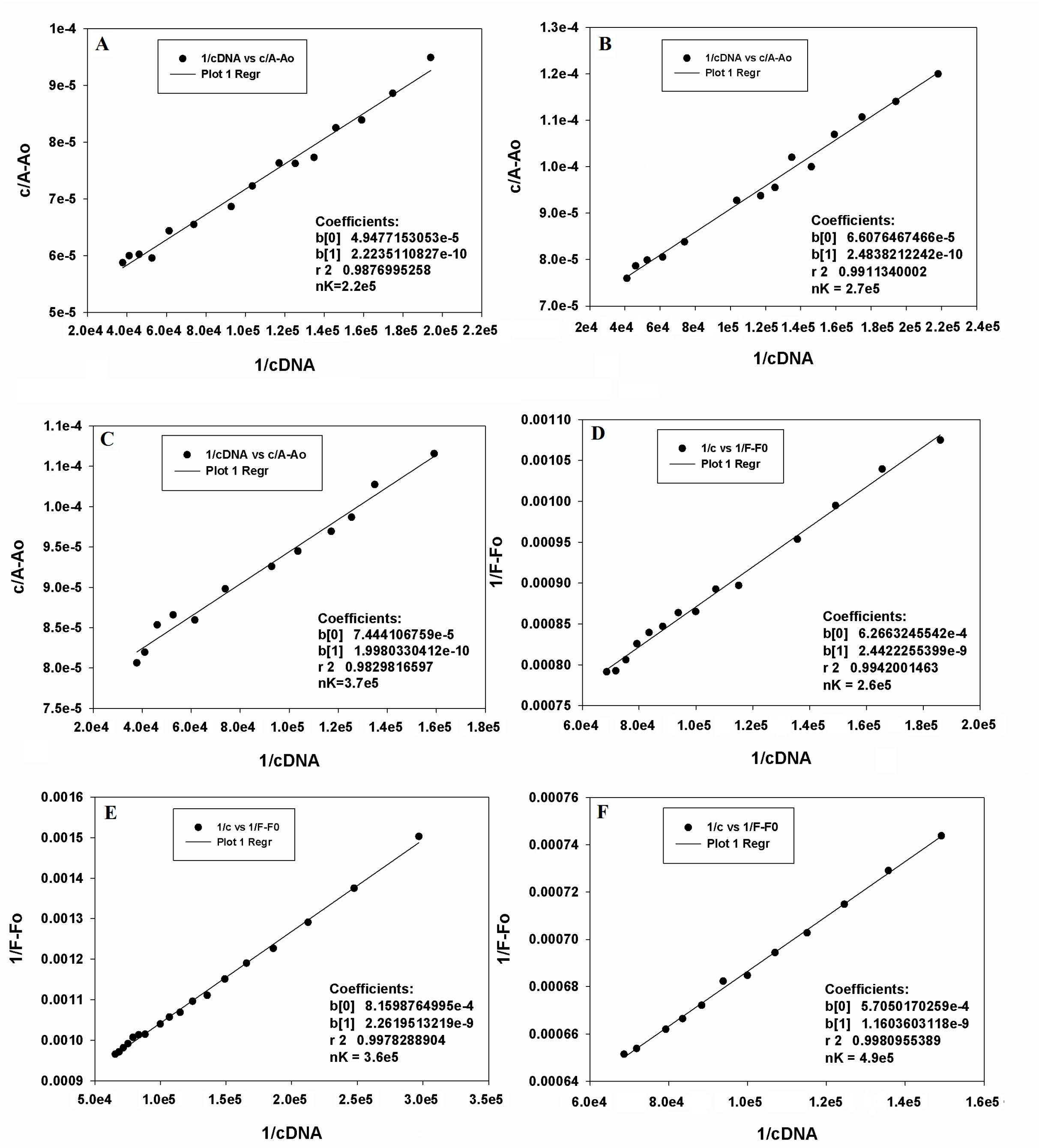


**Figure S3.** Benesi-Hildebrand plots of absorbance (A,C,E) and fluorescence (B,D,F) binding data of ligands **1** (A,B), **2** (C,D), and **3** (E,F) with G4 Bcl-2 2F8U.
